# Multi-state Markov model for time to treatment changes for HIV/AIDS patients: a retrospective cohort national datasets, Ethiopia

**DOI:** 10.1186/s12879-024-09469-9

**Published:** 2024-06-24

**Authors:** Tsegaye Hailu Kumsa, Andargachew Mulu, Joseph Beyene, Zeytu Gashaw Asfaw

**Affiliations:** 1 Department of Statistics, Hawassa University, Hawassa, Ethiopia; 2 Armauer Hansen Research Institute (AHRI), Addis Ababa, Ethiopia; 3 Faculty of Health Sciences, McMaster University, Hamilton, Canada; 4 Department of Epidemiology and Biostatistics, School of Public Health, Addis Ababa University, Addis Ababa, Ethiopia

**Keywords:** HIV/AIDS, ART, Multi-state survival model

## Abstract

**Background:**

Virological failure, drug resistance, toxicities, and other issues make it difficult for ART to maintain long-term sustainability. These issues would force a modification in the patient's treatment plan. The aim of this research was to determine whether first-line antiretroviral therapy is durable and to identify the factors that lead to patients on HAART changing their first highly active antiretroviral therapy regimen.

**Methods:**

A retrospective cohort study was conducted from October, 2019—March, 2020 across all regional states including Addis Ababa and Dire Dawa administrative cities. The target population is from all health facilities that have been providing ART service for at least the past 6 months as of October 2019. Multi-stage clustered sampling method was used to select study facilities and participants. Simple random selected ART medical records of patients ever enrolled in ART treatment services. We adopted a multi-state survival modelling (msm) approach assuming each treatment regimen as state. We estimate the transition probability of patients to move from one regimen to another for time to treatment change/switch. We estimated the transition probability, prediction probabilities and length of stay and factor associated with treatment modification of patients to move from one regimen to another.

**Results:**

Any of the six therapy combinations (14.4%) altered their treatment at least once during the follow-up period for a variety of reasons. Of the patients, 4,834 (13.26%) changed their treatments just once, while 371 (1.1%) changed it more than once. For 38.6% of the time, a treatment change was undertaken due to toxicity, another infection or comorbidity, or another factor, followed by New drugs were then made accessible and other factors 18.3% of the time, a drug was out of supply; 2.6% of those instances involved pregnancy; and 43.1% involved something else. Highly active anti-retroviral therapy (HAART) combinations TDF + 3TC + NVP, d4T + 3TC + NVP, and TDF + 3TC + EFV were high to treatment alterations in all reasons of treatment modifications, with 29.74%, 26.52%, and 19.52% treatment changes, respectively. Early treatment modification or regime change is one of the treatment combinations that include the d4T medication that creates major concern. The likelihood of staying and moving at the the start of s = 0 and 30-month transitions increased, but the likelihood of staying were declined. For this cohort dataset, the presence of opportunistic disease, low body weight, baseline CD4 count, and baseline TB positive were risk factors for therapy adjustment.

**Conclusion:**

Given that the current study took into account a national dataset, it provides a solid basis for ART drug status and management. The patient had a higher likelihood of adjusting their treatment at some point during the follow-up period due to drug toxicity, comorbidity, drug not being available, and other factors, according to the prediction probability once more. Baseline TB positivity, low CD4 count, opportunistic disease, and low body weight were risk factors for therapy adjustment in this cohort dataset.

**Supplementary Information:**

The online version contains supplementary material available at 10.1186/s12879-024-09469-9.

## Background

Since the beginning of the HIV/AIDS epidemic treatment, HIV infection has changed from an incurable illness to a manageable disease with a life expectancy approaching that of the general population in 2011 but updated in 2022 [1]. According to global fact sheet, In 2021, around 650, 000 people died from AIDS-related illnesses worldwide, compared to 2.0 million people in 2004 and 1.4 million people in 2010. According to a single point gauge, in Ethiopia there were approximately 11,627 annual death in all age in 2021 and declined to 10,421 annual death in all age in 2022 [2]. Globally, 28.7 million people living with HIV were receiving antiretroviral treatment (ART) in 2021and the coverage was 75% in 2021 [3] and also in Ethiopia increased from 3% in 2005 to 56% in 2014 and 78% in 2020 [4]. But, due to unique individual circumstances, patients have undoubtedly taken a variety of medications during ART follow-up periods. The most popular ART available in Ethiopia medications are abacavir (Ziagen), emtricitabine (Emtriva), lamivudine (Epivir), tenofovir disoproxil fumarate (Viread), and zidovudine (Retrovir). Despite the fact that these medications greatly improve the health of HIV/AIDS patients, difficulties with sedative toxicity and the complexity of current ART regimens continue to be a major source of worry [5]. Patients typically continue using treatments inconclusively since ART does not completely eradicate the virus once it has started [5].

However, enabling long-term sustainability is now highly active antiretroviral therapy (HAART's) new challenge. Many patients will be obliged to alter or adjust their therapies or regimens for a variety of reasons, including toxicity, comorbidity, pregnancy, or therapy failure. Large- and small-scale studies from developed and developing countries have shown that a substantial number of patients (up to 70%) may modify their regimen overtime, where 19.6%—44% of them modify their initial treatment within the first years of treatment [6–10]. In Ethiopia, only a few literatures were published and also, they are limited to specific hospitals and regions. With this end, national level analysis is very timely and important for various reasons such as; to import relevant drug, for planned drug distribution among HIV/AIDS patient caring hospitals so that address timely for those who are at need and to implement national level intervention.

According to recent WHO recommendations, d4T should be replaced with TDF and AZT wherever possible in first-line standard therapy. However, the switch from d4T to TDF has been gradual due to cost and toxicant management within limited resources setting [11]. In order to secure much higher well-being boosts, patients may therefore modify the sedative; of course, people may change more than one medication. Individuals in this study therefore go through a variety of events, as well as many intermediates and end points. This is typically accomplished by performing distinct analyses for each end point as well as for the intermediate events, but this is unsatisfactory because it ignores the connections between these events.

Therefore, the aim of this research was to determine whether first-line antiretroviral therapy is durable and to identify the factors that lead to patients on HAART changing their first highly active antiretroviral therapy regimen in Ethiopia and heading to support national health policies. This paper adopted a multi-state survival modelling (MSM) [12] supposing each treatment or regimen as state. We estimate the transition probability, length of stay and prediction of initial commencement of treatment of patients to move from one regimen to another in general as well as due to a specific event that generates the move. The model allows modelling of the occurrence of different event types (such as, single drug substitution or regimen switch) and the occurrence of subsequent events. The predictive models for multi-state data were also measured.

## Materials and Methods

### Data description

A Health facility based a retrospective cohort study was conducted from October, 2019—March, 2020 across all regional states including Addis Ababa and Dire Dawa administrative cities. Individuals were randomly selected from patient history medical records to determine ART patient treatment outcomes. Patients presenting with WHO stage 3& 4, a CD4 count below < = 350 and viral load greater than 1000 copies/ml were eligible to start ART [11]. We have considered demographic characteristics (age, weight, haemoglobin, sex, marital status, educational level, region, type of health facility), type of ART regimen initiated, patterns of ARV drug regimen change, and level of adherence. Patients medical records was also checked for presence of any opportunistic infection at base line and during ART, liver enzyme level, Hepatitis B and C serology status, and counselling related to family planning. In addition, CD4 cells count and HIV viral load were measured at each visit. Also decisions on which treatment regimen to start or substitute are made by the clinician based on immunological, viral load and other clinical diagnosis. During the study period, the standard treatments were abacavir (Ziagen), emtricitabine (Emtriva), lamivudine (Epivir), tenofovir disoproxil fumarate (Viread) and zidovudine (Retrovir). All the data, including demographic, clinical condition, laboratory results, and medication were recorded and entered to the central database using RedCap software. Out of the total, drug information missed for 5824 patients and excluded from this analysis and a total of 33,716 were included in the final data analysis.

### Study population

All medical records of HIV/AIDS patients who began and were enrolled in anti-retroviral therapy at all health facilities in Ethiopia made up the source population for this study (record review). As of October 2019, all medical facilities that have been offering ART services for at least the previous six months are included in the target population.

### Sampling techniques

The region was the area of analysis, and a nationally representative participants and a multi-stage cluster sampling method was used to select the study facilities and participants. First, 63 hospitals and their two catchment health centrs were sampled. This was later proportionally allocated to the nine regions and two city administrations. In each region, hospitals with ART caseloads of 200 patients or more and their corresponding catchment health centers with an ART caseload of 100 patients were randomly (simple random/lottery method) selected. Available dataset were used from the study which calculated sample size was initially distributed to each region proportionally. Then, the allocated sample size in each region was later on distributed to the selected hospitals following a proportional allocation. Finally, the allocated sample size in each selected health facility was again proportionally distributed to the four ART starting strata and time periods: January 21, 2011 – December 31, 2013 (CD4 count < 200), January 01, 2014 – December 31, 2014 (CD4 count < 350), January 01, 2015 – December 31, 2016 (CD4 count < 500) and January 01, 2017 – December 31, 2018 (the test and treat approach).

### Sample size determination

This study used of the dataset that was made available by the previous research, which reviewed the charts of nearly 39,590 ART patients from October, 2019—March, 2020. The sample size of the study was calculated based on the result of the study conducted 2011 nationaly and considerations included 90% power and a 95% degree of confidence (= 0.05). Furthermore, 50%, 17% of lost follow-ups, 10% of deaths, and 95% correlation were considered as hazard ratios. A design effect of 2 was also considered, as the health facility was chosen using a multistage clustered sampling technique. These theories led to the determination of a sample size of 37,000 patients in total, and the examination of 39,590 medical records.

#### Outcome variable and operational definition

The main outcome of this study is “time–to–treatment change (treatment modification or regimen switching)” in which treatment change is defined as changing at least one of ART in the regimen without initiating a second line therapy. However, dosage modifications of any combination were not considered as treatment change.

***Switch:*** A discontinuation of a failed any-line ART regimen and the start of a new combination of treatment either of the treatment availability based on the clinician decision.

***Event:*** A switch or shift of treatment because of any other reasons like drug interaction, side effect, toxicity and others.

***Censored:*** Includes lost to follow up, transferred out, and dead before switched to any-line ART within the follow up period and, those patients who did not switch at the end of follow up.

***Survival time:***Time from confirmed current treatment failed to switch to any-line of ART or censored measured in days till the end of follow-up (84 month).

#### Multi-state model

### Model formulation

There were 22 ART regimen combinations, however only nine of them were first line ART regimen combinations, accounting for 99.64% of the total. These are 1a: d4T + 3TC + NVP, 1b:d4T + 3TC + EFV, 1c: AZT + 3TC + NVP, 1d: AZT + 3TC + EFV, 1e: TDF + 3TC + EFV, 1f: TDF + 3TC + NVP, 1 g: ABC + 3TC + EFV, 1 h: ABC + 3TC + NVP, 1j: TDF + 3TC + DTG. However, the six line regimens had been started by 99.2% of the individuals.

**Fig. 1:**
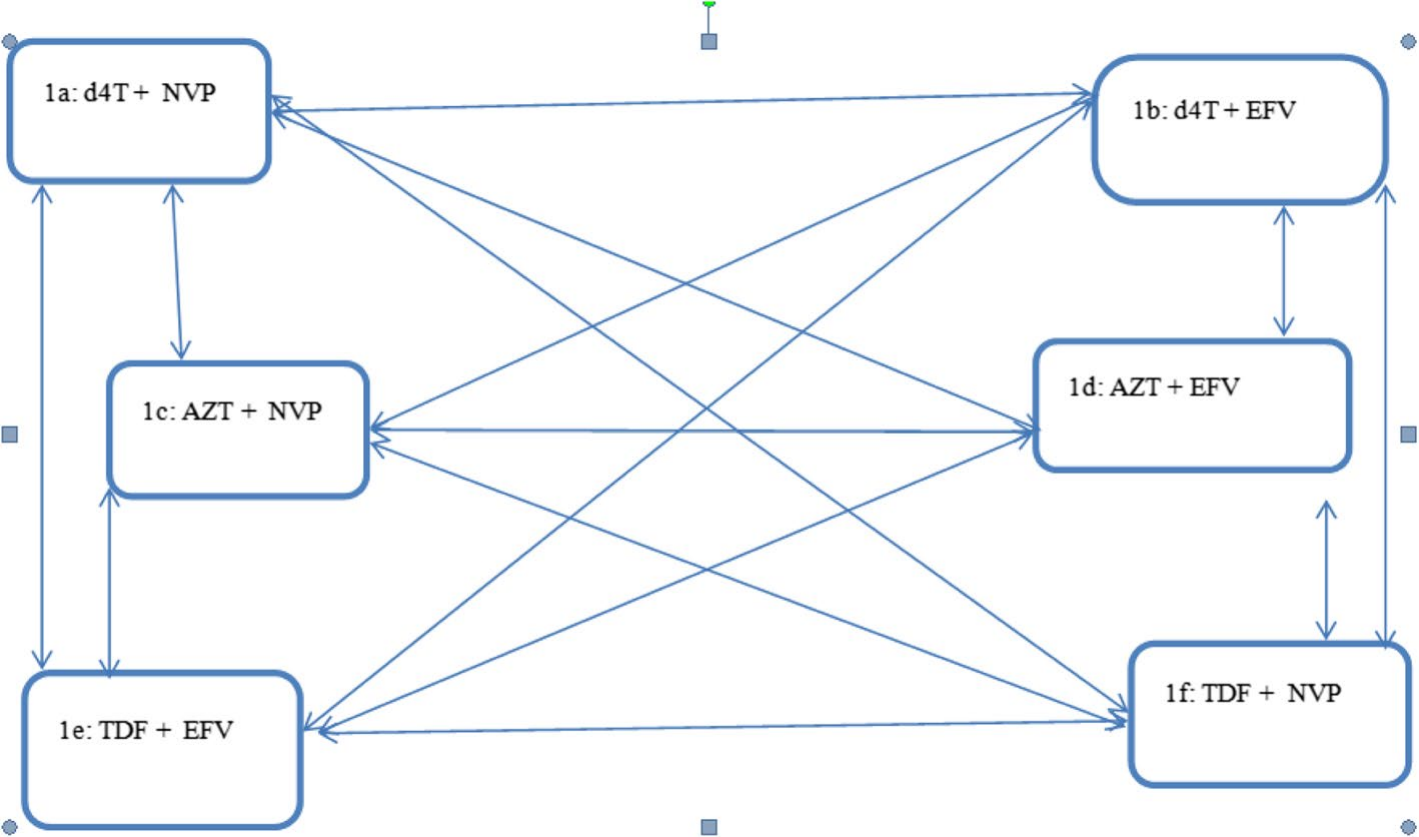
A Six-state multi state model for treatment change. 3TC was ignored because it was present in all the regimens

Figure 1 shows the treatment history of individuals receiving ART and shows that there may have been a shift between medication combinations for a variety of reasons. The word "state" will henceforth refer to a particular set of treatments. The model makes the assumption that every patient can at some time switch to the whole regimen and that it is reversible. The six transitory "states" of the model are as follows: d4T + 3TC + NVP (1), d4T + 3TC + EFV (2), AZT + 3TC + NVP (3), AZT + 3TC + EFV (4), TDF + 3TC + EFV (5), and TDF + 3TC + NVP (6). These six first-line therapy combinations are represented by these states.

A patient at the first “state” may be at risk or possible to transit from 1a—> 1b or 1a—> 1c, or 1a- > 1d or1a—> 1f or 1a—> 1e, this subject is named as “single drug-substitution” (treatment modification). Transition from 1a to 1b implies that the patient has substituted their Non-nucleoside reverse transcriptase inhibitors (NNRTI’s(NVP and EFV) NVP by EFV without changing their nucleoside/nucleotide reverse transcriptase inhibitors (NRTIs) NRTI treatment (d4T, AZT or TDF). However, transition 1a to 1c implies that the patient has substituted their NRTI’s d4T by AZT without changing their (NNRTI) treatment. Transitions 1a to1d or 1a to 1e, imply regimen switching, substituting both NNRTI and NRTI at the same time. After making one of these possible transitions patients will be at risk of making further transition.

The multi-state model is an effective tool for describing a subject's transitions between treatments over time. For each potential transition, the model calculates the transition probabilities [13]. For the treatment switching data, this study developed a six-state continuous time homogeneous multi-state Markov model. If the future of a process depends exclusively on the present, the process is Markovian [14]. Figure 1 shows the model in a visual manner.

The potential transitions are shown by the arrows. The double-sided arrows suggest reversible changes. If the subject switches back and forth between two states, the transitions are reversible.

A continuous time stochastic process model that permits people to choose between a limited number of states is known as a multi-state model (MSM) [15]. If the process is in state ℓ^th^ at time t (in months), then the stochastic process (X_t_, t > 0) is defined as X_t_ = ℓ [15]. As was already mentioned, there are six therapy changes for the case study discussed in this work, which suggests that the patient's beginning condition was X $\in (1\text{a}, 1\text{b}, 1\text{c},1\text{d},1\text{e},1\text{f})$. Modelling the change from the ℓ^th^ regimen (state ℓ) to the j^th^ regimen (state j) at time t is our main goal. The transition intensities, or hazard rate, a_ℓj_(t), which reflects the instantaneous risk of a transition from state into state j at time t, that is, the distribution of this multi-state process [16].

1 $$\begin{array}{cc}\underset{\Delta\text{t}\rightarrow0}{a_{\ell\text{j}}(\text{t})=\text{lim}}\frac{\text{p}({\text{X}}_{\left(\Delta\text{t}+\text{t}\right)}=\text{j}\vert{\text{X}}_\text{t}=\ell,{\text{ F}}_{\text{t}-})}{\Delta\textrm{t}}\ell,&\text{j}\in(1\text{a},1\text{b},1\text{c},1\text{d},1\text{e},1\text{f}),\ell\neq\text{j}\end{array}$$

Here, F_t−_ represents process history prior to time t. In our application, time t represents time since ART initiation. The cumulative transition hazard is defined as:

A_ℓj_(t)=${\int }_{0}^{t}{\alpha }_{{\ell}j}(u)du, \left(u\le t\right), where {A}_{{\ell}j}\left(t\right)=0$ if a direct transition between state ℓ and j is impossible. These intensities can be gathered in to a 6 × 6 matrix A(t) with diagonal elements.

$${\text{A}}_{{\ell}{\ell}}\left(\text{t}\right)\hspace{0.17em}=\hspace{0.17em}-\sum_{j=1, {\ell}\ne \text{j}}^{6}{A}_{{\ell}j}\left(t\right), {\ell},\text{j}\hspace{0.17em}=\hspace{0.17em}\{1\text{a},1\text{b},1\text{c},1\text{d},1\text{e},1\text{f}\}$$

#### Markov models

Assuming that the information presented up to this point is accurate, the main concern with ART delivery is the possibility of the patient's subsequent therapy regimen (patient status). In the case of a patient who switched from d4T to AZT after six months without changing his NNRTI and (i.e., the patient's current state is either in states, depending on the initial NNRTI component) who had no additional events at one-year post-ART, one might be concerned about determining the likelihood of remaining on this grouping for an additional six months as well as associating this likelihood to a patient who did not supermom their NRTI (d4T). In this work, we used transition probabilities to predict a patient's status over the long term. Let s be the moment at which the prediction is made measured from the patient's time origin (the beginning of therapy), and let's use *X*_*u*_,0 ≤ *u* ≤ *s*.to represent the patient's event history up to time s. Given the information available up until time s, the transition probability from state l to state j in the time period [s, t] is then defined as

2 $$P_{lj}\left(s,t\right)=P\left(X_t=j\vert X_s=\ell,X_u\right),s\leq t,\ell,j\in\left\{1a,1b,1c,1d,1e,1f\right\}u\in\left[0,s\right]$$

Markov model appropriate to estimate *P*_*ℓj*_(*s*,*t*) [17]. The model undertakes the next status of the patient depends on the current time. The past history of the patient has effect on the risk. This indicates that.

3 $$\alpha_{\ell j}(t)\;\mathrm{dt}=\;P\left(X_{(t+\Delta t)}-=j\vert X_t-=\ell\right),\ell,j\in\left\{1a,1b,\dots,1f\right\},\ell\neq j$$

4 $${P}_{{\ell}j}\left(s,t\right)=P\left({X}_{t}= j|{X}_{s}={\ell}\right), s\le t,{\ell},j \in \left\{1a, 1b, \dots , 1f\right\}$$

Similar to **A***(t)*, these probabilities can be assembled in to a 6 × 6 matrix **M***(s*, *t)* with ${P}_{{\ell}j}\left(s,t\right)$ as its *(ℓ*, *j)*^*th*^ entry. A single element ${P}_{{\ell}j}\left(s,t\right)$ combines both direct and indirect transition from state *ℓ* to state j [17].

#### Estimation

Combinations of transition-specific survival models can be used to specify a multistate model. Using start and stop notation, a patient has a row of data for each transition for which they are at risk. After ART begins, patients who have no transition should continue receiving their initial treatment (starting state). When there is no feasible transition from ℓ to j, there is zero probability of leaving this state, which is represented by the entry (ℓ, j). Each row's elements in the transition matrix add up to zero. Finding the unidentified transition intensities that optimize the likelihood is the process of fitting a multi-state model.

The mean stopover time in each state, which represents the typical amount of time a patient spends in a transient state during a single stay before travelling to other states, is estimated by the multi-state model. The expected stopover time is calculated as -1/λ_jj_, where λ_jj_ is the j^th^ diagonal entry of Q(t) [18].

$$Q(t)=\left[\begin{array}{cccccc} -\lambda11 & -\lambda12 & -\lambda13 & -\lambda14 & -\lambda15 & -\lambda16\\ -\lambda21 & -\lambda22 & -\lambda23 & -\lambda24 & -\lambda25 & -\lambda26\\ -\lambda31 & -\lambda32 & -\lambda33 & -\lambda34 & -\lambda35 & -\lambda36\\ -\lambda41 & -\lambda42 & -\lambda43 & -\lambda44 & -\lambda45 & -\lambda46\\ -\lambda51 & -\lambda52 & -\lambda53 & -\lambda54 & -\lambda55 & -\lambda56\\ -\lambda61 & -\lambda62 & -\lambda63 & -\lambda64 & -\lambda65 & -\lambda66 \end{array}\right]$$

as [19].

$$P(t) = exp[Q(t)]$$

The transition probability matrix is given by:

$$\text{P}(\text{t})=\left[\begin{array}{cccccc} p11 & p12 & p13 & p14 & p15 & p16\\ p21 & p22 & p23 & p24 & p25 & p26\\ p31 & p32 & p33 & p34 & p35 & p36\\ p41 & p42 & p43 & p44 & p45 & p46\\ p51 & p52 & p53 & p54 & p55 & p56 \\ p51 & p52 & p63 & p64 & p65 & p66\end{array}\right]$$

The row sum of P is equal to one, For the absorbing state j, P_jj_(s,t) = 1.

The likelihood function is formed with the transition probabilities. This likelihood function, L(β)is given by,

$$L\left(\upbeta \right)=\prod_{{\ell}\text{j}}{\text{L}}_{{\ell}\text{j}}=\prod_{{\ell}\text{j}}{\text{P}}_{\text{s}\left({\text{t}}_{{\ell}\text{j}}\right)\text{s}\left({\text{t}}_{{\ell},\text{j}+1}\right)}({\text{t}}_{{\ell},\text{j}+1}-{\text{t}}_{{\ell}\text{j}})$$

Where ${\text{L}}_{{\ell}\text{j}}$ is the entry of the s(tℓj)th row and s(tℓ,j + 1)th column in the transition probability matrix [20].

#### Semi-parametric estimation

### Multi-state regression models

Models for multiple-state regression X_i_(t) is defined as the equation in (1). We are presuming that all subjects will experience the same intensities. However, in real-world scenarios, it can be helpful to use a covariate vector, Z, which may be time-dependent, to connect the individual characteristics to the intensity rates. For a general regression model we can write. Generally Multi-state regression models can be written as ${\alpha }_{{\ell}\text{ji}(.)}=\varphi ({\alpha }_{{\ell}\text{j}.0\left(.\right),}{\upbeta }_{{\ell}\text{j}}^{\rm{T}}{\text{Z}}_{i})$ [21], Where ${\alpha }_{{\ell}\text{j}.0\left(.\right) }$is the baseline intensity function between states ${\ell}$ and j. β_ℓj_ is the vector of regression parameters, and Z_i_ is the covariate vector for subject i.

Similar to the survival model, the proportional hazards regression model was used to incorporate the covariate effects on transition intensities [22]. Given a covariate vector Z,

$${\varvec{\lambda}}\left({\varvec{t}}|{{\varvec{Z}}}_{{\ell}{\varvec{j}}{ }}\right)={{\varvec{\lambda}}}_{{\ell}{\varvec{j}}0{ }}{\mathbf{e}\mathbf{x}\mathbf{p}{ }[{\varvec{\beta}}}_{{\ell}{\varvec{j}}}^{{\varvec{T}}}{ }{{\varvec{Z}}}_{({\varvec{t}})}]$$

Β_ℓj_ is the vector of regression coefficients and ${{\varvec{\lambda}}}_{{\ell}{\varvec{j}}0}$ is the baseline hazard. We used the package msm in R version 4.3.0 to perform the multi-state analysis. The result of the multi-state model to obtain predictions at a certain time after treatment modification for patients with a given set of covariates and a given set of pro- treatment switching event. The model selection was done with the likelihood ratio test [17].

## Results

### Socio-demographic characteristics

This national based retrospective cohort study intended to model treatment switching of patients receiving ART during their follow-up period in their health facility. Out of the total of 39,590 patients, 33,716 were included in this analysis and the remaining excluded by missing information about the treatment drug. Tables 1 and 2 below shows demographic and clinical related characteristics of the patients.

**Table 1:** Socio-demographics characteristics (*N* = 33,716)

Variable	Number (%)
Sex	
Male	13,200(39.18%)
Female	20,490(60.82%)
Age Group	
< = 20	1,011(3%)
21–30	7,474(22.17%)
31–40	13,249(39.30%)
41–50	8,149(24.17%
51–60	2,876(8.53%)
> = 61	953(2.84%)
Age(mean)	37.79 ± 10.37935
Marital status	
Single	5654(16.78%)
Married	15,812(46.94%)
Divorced	6,510(19.32%)
Widowed	2,648 (7.86%)
Not specified	3,064(9.10%)
Educational status	
Illiterate	8109(24.07%)
Primary	**10,589(31.43%)**
Secondary	8,220(24.4%)
Tertiary	**3,509 (10.42%)**
Not specified	3,260 (9.68%)

**Table 2:** Baseline clinical characteristic’s (*N* = 33,716)

WHO clinical stages of	
HIV/AIDS	
Stage 1	12,786 (38%)
Stage 2	7,855(33.35%)
Stage 3	9,884 (29.38%)
Stage 4	2,589(7.7%)
Not specified	531(1.6%)
Opportunistic infection	15,145 (44.92%)
Not OI	
OI	10,647(31.6%%)
Not Specified	7,924(23.5%)
Baseline status	29,007(87.0%)
Working	
Ambulatory	3,074(9.2%)
Bedridden	749(2.3%)
Not specified	500(1.5%)
History of Adherence	
Good	30,132(93.1%)
Fair	80(00.25%)
Poor	384(1.2%)
Not specified	1,774(5.5%)
TB Status	8,791 (26.1%)
Positive	
Negative	24,925(73.9%)
Baseline CD4 + cell: median(IQR)	220(232)
Baseline Regimen	
1a (d4T + 3TC + EFV)	193(0.6%)
1b(d4T + 3TC + NVP)	21(0.2%)
1c (AZT + 3TC + EFV)	2972(8.9%)
1d (AZT + 3TC + NVP)	1,534 (4.6%)
1e(TDF + 3TC + NVP)	26,404(79.2%)
1f (TDF + 3TC + NVP)	2208(6.6%)

The average follow-up time was 37.27 ± 24.00 months per person. The majority of the participant were women 20,490(60.82%) and 13,200(39.18%) of the patients were men, according to WHO fact sheet, in 2021 majority of the patient were Female than Male. The mean age of the participant was 37.8 ± 10.37 years and 3829(11.17%) were patients 50 years and older. Majority of the patients were around average age between 21 to 40 years old 20,723(63.47%).This indicated that most productive age group were affected by this disease and giving treatment is mandatory. Delivering appropriate information to the Ministry of heath helps to prepare good policy and saving this fertile age group. At ART initiation, patients had a median CD4 cells count of 220 cells/mm3 (IQR: 119 -351 cells/mm3) (Table 3). Majority of the patients were WHO stage 2,3 & 4 (60.4%), followed by WHO stage one 12,786 (38%) and according to WHO recommendation in 2021, majority of the patients fulfilled to start the ART treatment. This nearly half of the patients were married 15,852(46.94%), followed by divorced 6,510(19.32%) and never married 5654(16.78%). Educational status of the participants were illiterate and primary school 18,698 (55.5%), followed by 8,220(24.4%) and very few were 3,509 (10.42%).

**Table 3:** Summary of the transitions matrix

	1a	1b	1c	1d	1e	1f	Total
1a	*	4	92	4	19	21	140
		(2.86%)	(65.71%)	(2.86%)	(13.57%)	(15%)	(100%)
		(23.53%)	(51.4%)	(5.06%)	(2.08%)	(4.88%)	
1b	1	*	8	20	23	3	55
	(1.81%)		(14.54%)	(36.36%)	(41.81%)	(5.45%)	100%)
	(8.33%)		(4.49%)	(25.32)	(2.52%)	(0.69%)	
1c	6	6	*	95	179	98	384
	(1.56%)	(1.56%)		(24.74%)	(46.61%)	(25.53%)	(100%)
	(50%)	(35.29%)		(55.23%)	(19.61%)	(22.79%)	
1d	1	3	28	*	140	18	190
	(1.81%)	(0.17%)	(14.73%)		(73.68%)	(9.47%)	(100%)
	(8.33%)		(15.64%)		(15.33%)	(4.18%)	
1e	4	4	42	50	*	290	
	(1.02%)	(0.23%)	(10.77%)	(15.82%)		(74.36%)	390
	(33.33%)	(23.53%)	(15.64%)	(29.07%)		(67.44%)	(100%)
1f	0	0	9	3	552	*	564
	(0.0%)	(0.0%)	(1.59%)	(0.53%)	(97.87%)		(100%)
			(5.02%)	(0.17%)	(60.46%)		
Total	12(100%)	17(100%)	179(100%)	172(100%)	913(100%)	430(1005)	1723

Note: 1a: d4T + 3TC + NVP, 1b: d4T + 3TC + EFV, 1c: AZT + 3TC + NVP, 1d: AZT + 3TC + EFV, 1e: TDF + 3TC + EFV, and 1f: TDF + 3TC + NVP

A total of 22 ART regimens were used by 33,660 study participants. About (99.64%) were on nine of first line ART regimens:1a:d4T + 3TC + NVP, 1b:d4T + 3TC + EFV, 1c:d4T + 3TC + NVP, 1d:AZT + 3TC + NVP, 1e:TDF + 3TC + EFV, 1f:TDF + 3TC + NVP, 1 g:ABC + 3TC + NVP, 1j and 1 h. Less than 0.5% of them was on second line (0.34%) and third lines ART regimens (0.02%). However, 99.2% of the participants were on six first line regimens: 1e-TDF + 3TC + EFV (n = 26,404; 78.44%), 1c-AZT + 3TC + NVP (n = 2,972; 8.83%), 1f-TDF + 3TC + NVP (2,208; 6.56%), 1d-AZT + 3TC + EFV(n = 1,534; 4.56%),1a-d4T + 3TC + NVP(193; 0.57%), and 1b-d4T + 3TC + EFV (81; 0.24%). The majority of the patients (85.64%) had no treatment modification, 4,463 patients (13.26%) had their treatment changed only once while 371 patients (1.11%) had their treatment changed more than once.

### Descriptive statistics analysis

#### Cumulative probability of treatment modification

**Fig. 2:**
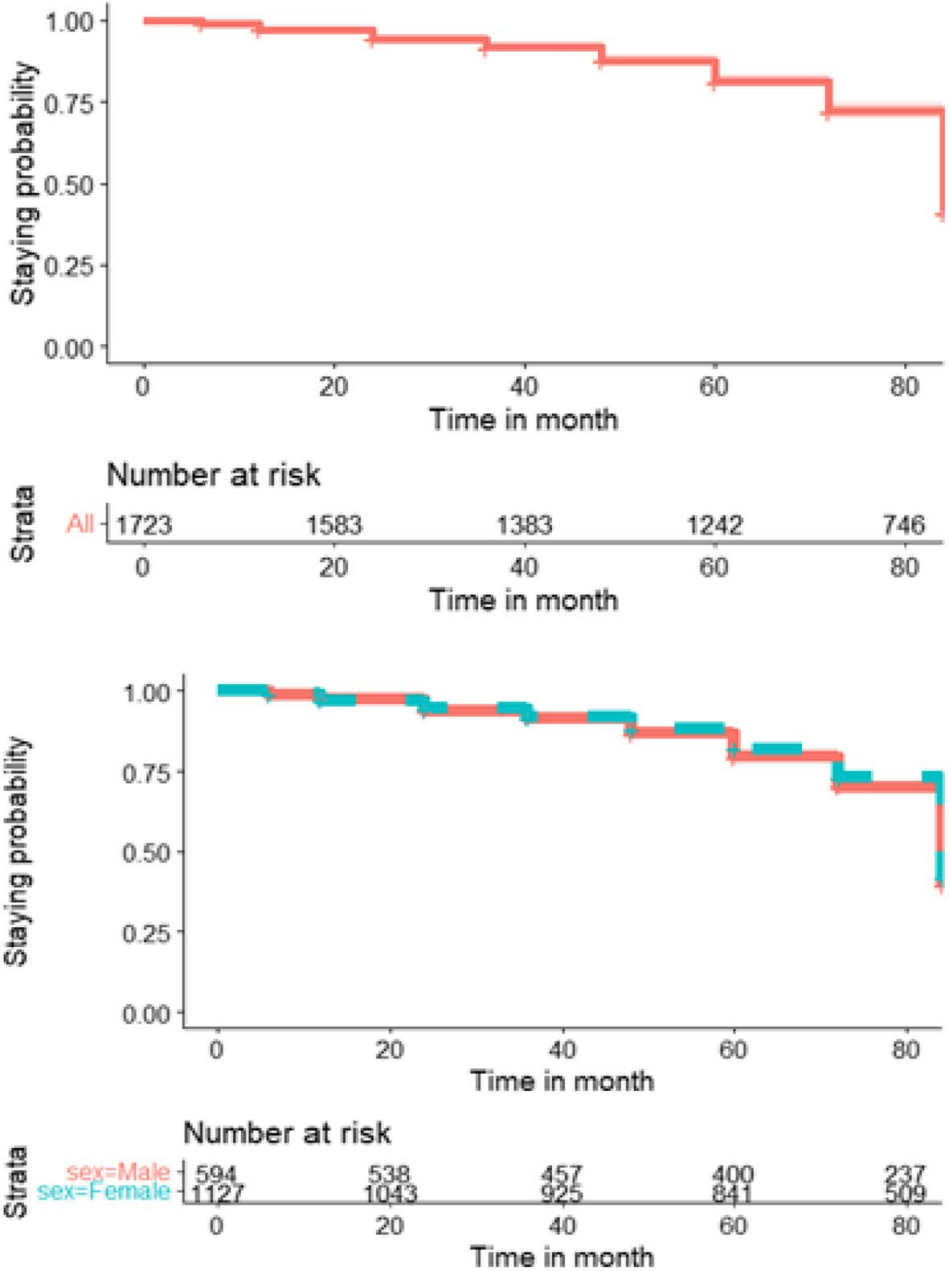
Overall staying probability and overall staying probability by sex. Note that high probability means better not modified treatment

The overall cumulative probability of ART treatment modification as time goes was lower and likely all patients to modify. Cumulative probability of staying (before modifying their medication) of the patient at each follow-up times 6, 12, 24, 36, 48, 60, 72 and 84 months were 0.91, 0.831%, 0.708%, 0.598%, 0.512%, 0.414%, 0.213 and 0.096% respectively. Similarly, the cumulative treatment modification probability by Kaplan–Meier survival curve [Fig. 2] showed that the time goes to end of treatment period, the probability of modified their treatment increased. These indicated that majority of the patients modified their treatments at the end of 84 months’ time follow-up period. Also the treatment modification probability between male and female patients shown Fig. 2 below, female patients’ had better not modified their treatment modification as compare to male.

##### Estimated Transition matrix

The summary of transition matrix of the process is shown in Table 3, from the total treatment modify was observed patients initiated on 1a:d4T + 3TC + more likely to modified to 1c:AZT + 3TC + NVP with 92(65.71%) out of total modification 140(8.13%).Also 1b:d4T + 3TC + EFV more likely to transit to 1e:TDF + 3TC + EFV 23(41.81%) out of a total transition 55(3.19%), The patient initiated 1c:AZT + 3TC + NVP more likely to transit to 1e:TDF + 3TC + EFV with 179(10.39%) out of 384((22.28%), The patient modified their treatment from 1d:TDF + 3TC + NVP to 1e:TDF + 3TC + EFV with 140(73.68%) out of the total 190(11.03%), from 1e:TDF + 3TC + EFV transit to 1f:TDF + 3TC + NVP with 290(74.36%)%) and from 1f:TDF + 3TC + NVP to 1f:TDF + 3TC + NVP were 552((97.87%) out of the total 564( 37.72%) respectively.

**Fig. 3:**
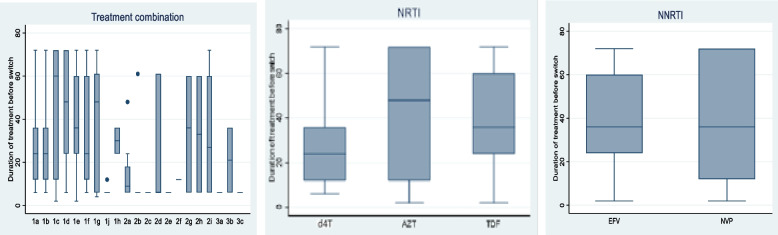
Duration in original treatment combination before switch, duration in original NRTI before switch, and duration in original NNRTI before switch NB: Note that only the time spent in the current treatment combination of the patients who modified their treatment are considered

The length of stay in each treatment combination before the first change to another treatment combination is presented in Fig. 3 above. As shown in the figure, when we look at the time spent in the current treatment combination of the patients who modified their treatment, patients initiated on AZT (42 months; IQR: 12–72) had a tendency to stay longer as compared to patient initiated on d4T (24 months; IQR: 12–36) and TDF (36 months; IQR: 24–60). Similarly, patients initiated on NVP had a tendency to stay nearly similar (36 months; IQR: 12–72) as compared to EFV (36 months; IQR: 24–72) but the IQR of NVP is larger than the IQR of EFV. It is interesting that regimens containing d4T were more prone to treatment modification than those containing AZT and TDF.

#### Reason for treatment switch

Table 4, From the total of 288(22.5%) patients modify their medication because of Toxicity/side effect were 1a was 23(1.8%), 1b was 11(0.86%), 1c was 90(7.04%), 1d was 31(2,43%), 1e was 45(3.5%) and 1f was 88(6.89%) respectively. Similarly, from the total of 202(15.78%) patients modify their medication because of others side effect ( drug out of stock, virology failure and other reason) were 1a was 11(0.86%), 1b was 6(0.46%), 1c was 69(5.39%), 1d was 24(1.87%), 1e was 45(3.45%) and 1f was 48(3.74%) respectively. The other new drug availability, Drug out of stock, other reason, other not specified reason effect were 27%, 5.11%, 12.6%, 6.91, 6.01% and 32.73% respectively.

**Table 4:** Reasons for antiretroviral modification among HIV patients on HAART

Reasons	**1a**	**1b**	**1c**	**1d**	1e	1f	Total
Toxicity/side effect	23(1.8%)	11(0.86%)	90(7.04%)	31(2.43%)	45(3.5%)	88(6.89%)	288(22.5%)
Pregnancy	0(0%)	1(0.08%)	10(0.78%)	5(0.39%)	3(0.23%)	4(0.31%)	23(1.8%)
Risk pregnancy	0(0%)	0(0%)	3(0.23%)	1(0.08%)	1(0.08%)	2(0.16%)	7(0.55%)
Due to new TB	2(0.16%)	0(0%)	17(1.33%)	1(0.08%)	5(0.39%)	12(0.94%)	37(2.9%)
Newdrug available	9(0.7%)	3(0.23%)	42(3.29%)	19(1.49%)	40(3.1%)	20(1.6%)	133(10.4%)
Drug out of stock	24(1.9%)	11(0.86%)	23(1.8%)	8(0.63%)	15(1.2%)	21(1.64%)	102(7.9%)
Other reason	6(0.47%)	5(0.39%)	20(1.56%)	9(0.7%)	14(1.1%)	22(1.7%)	76(5.95%)
Clinical treatment	0(0%)	0(0%)	5(0.39%)	3(0.23%)	8(0.63%)	3(0.23%)	19(1.49%)
Immunologic failure	2(0.16%)	0(0%)	4(0.32%)	2(0.16%)	0(0%)	1(0.08%)	9(0.7%)
Virology failure	1(0.08%)	0(0%)	10(0.78%)	3(0.23%)	13(1.02%)	4(0.32%)	31(2.43%)
Not specified	65(5.09%)	14(1.10%)	109(8.5%)	51(3.9%)	113(8.8%)	201(15.7%)	553(43.2%)

Note: 1a: d4T + 3TC + NVP, 1b: d4T + 3TC + EFV, 1c: AZT + 3TC + NVP, 1d: AZT + 3TC + EFV, 1e: TDF + 3TC + EFV, and 1e: TDF + 3TC + NVP

#### Transition probability

**Fig. 4:**
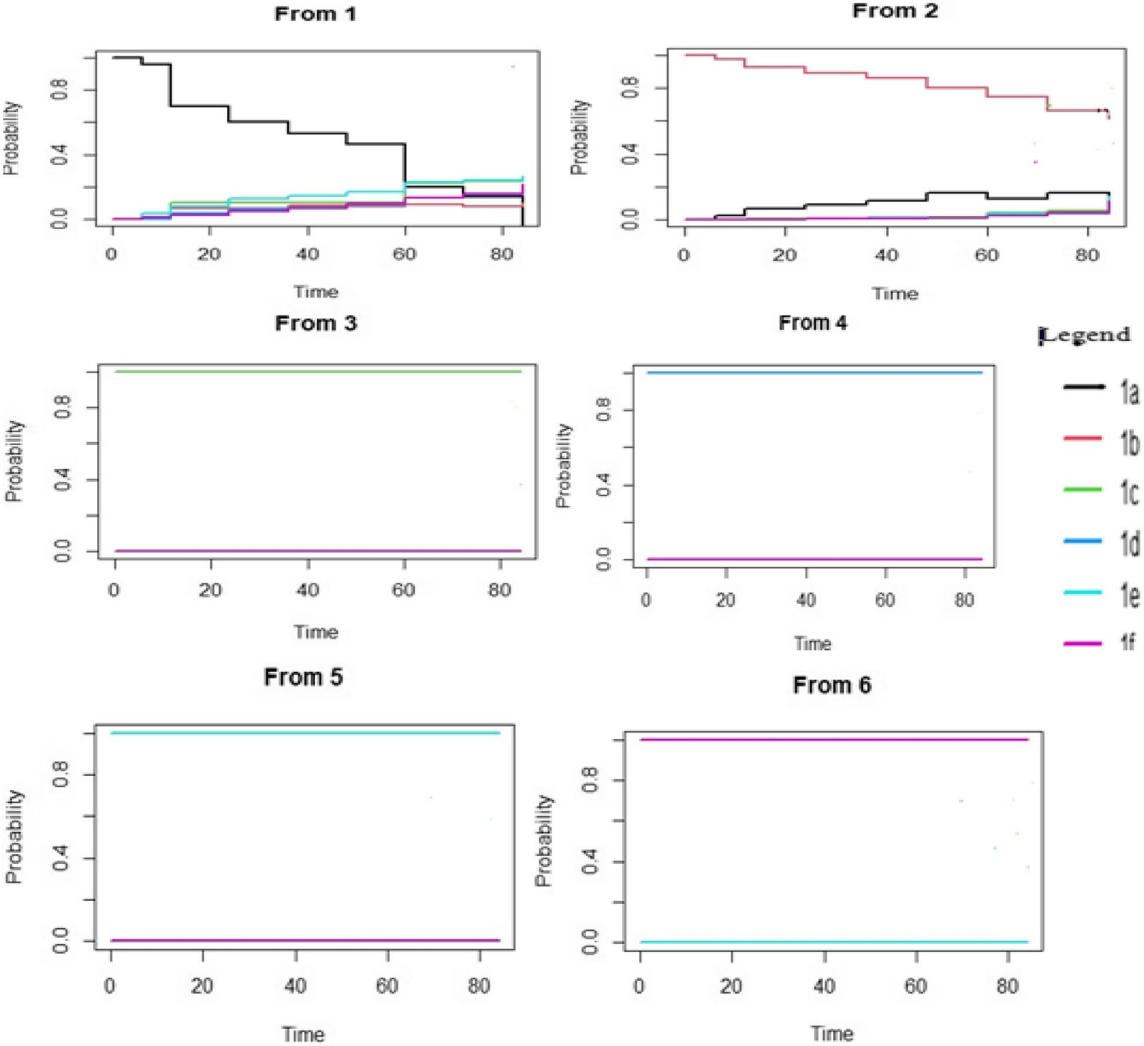
Transition probability starting from each state. Note: the estimate contains both direct and indirect transition probabilities. 1a: d4T + 3TC-NVP, 1b: d4T + 3TC + EFV, 1c: AZT + 3TC + NVP, 1d: AZT + 3TC + EFV, 1e: TDF + 3TC + EFV, 1f: TDF + 3TC + NVP

Using mstate package in R software [23] statistical analysis in transition probability starting from each state was calculated. The estimated transition probabilities are shown in Fig. 4 below, from all starting states to all possible states, between the starting time s = 0 and all event times successively is shown. Treatment combinations containing d4T have the lowest probability of treatment modification and as the follow up time went, the probability were decrease and high treatment modification observed. Initially at the start of ART d4T were lowest treatment modification but as the follow up time increase treatment modification were worst. AZT or TDF early the start of ART treatment worst treatment modification while as the follow up time increase the probability and lower the probability of treatment modification.

#### Prediction

**Fig. 5:**
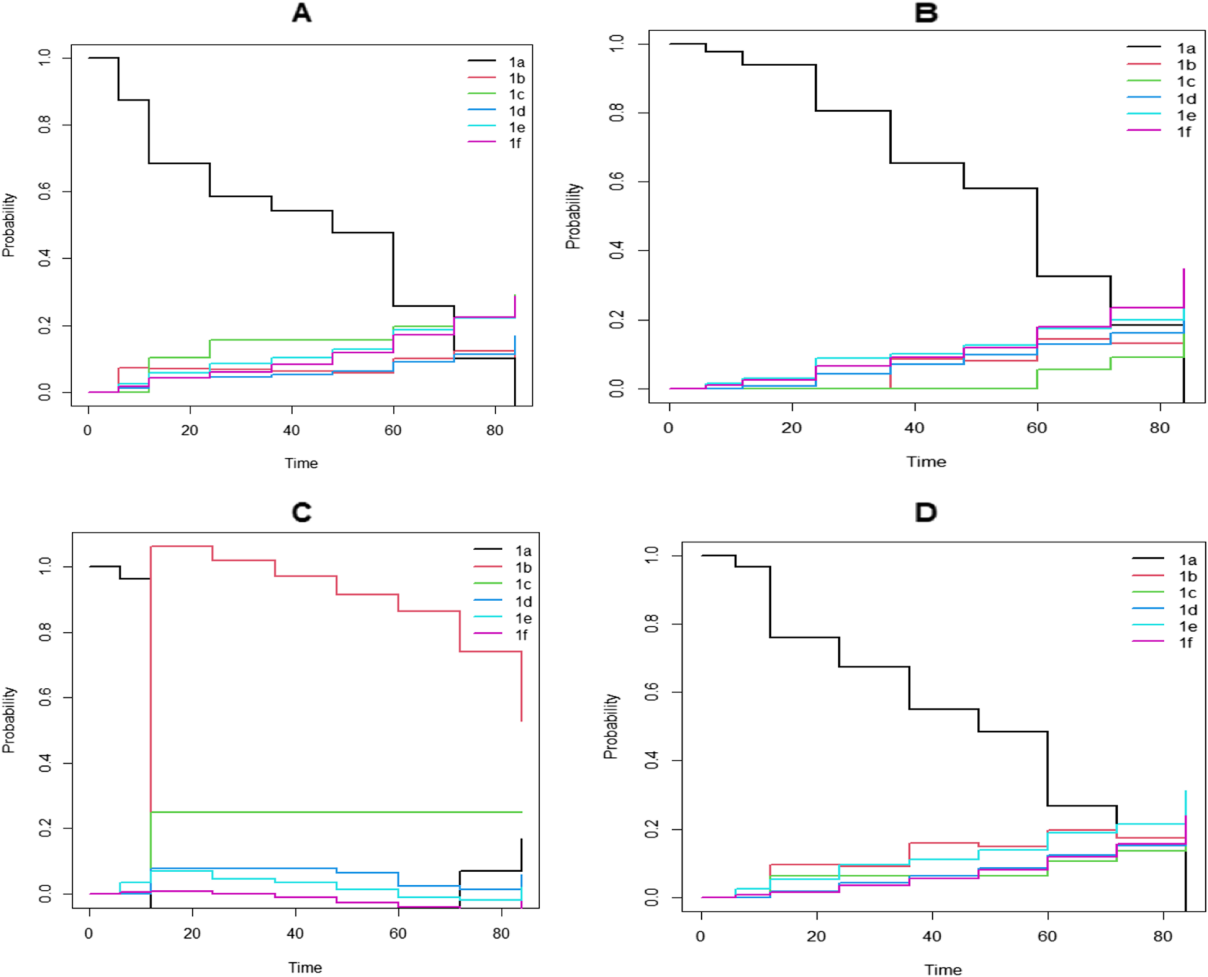
Prediction probabilities at s = 0 for a reference patient. **A** Probability of no treatment modification, **B.** Probability of NRTI substitution, **C.** Probability of NNRTI substitution and **D**. Probability of regimen changes. 1a: d4T-3TC-NVP, 1b: d4T + 3TC + EFV, 1c: AZT + 3TC + NVP, 1d: AZT + 3TC + EFV, 1e: TDF + 3TC + EFV, 1f: TDF + 3TC + NVP

**Fig. 6:**
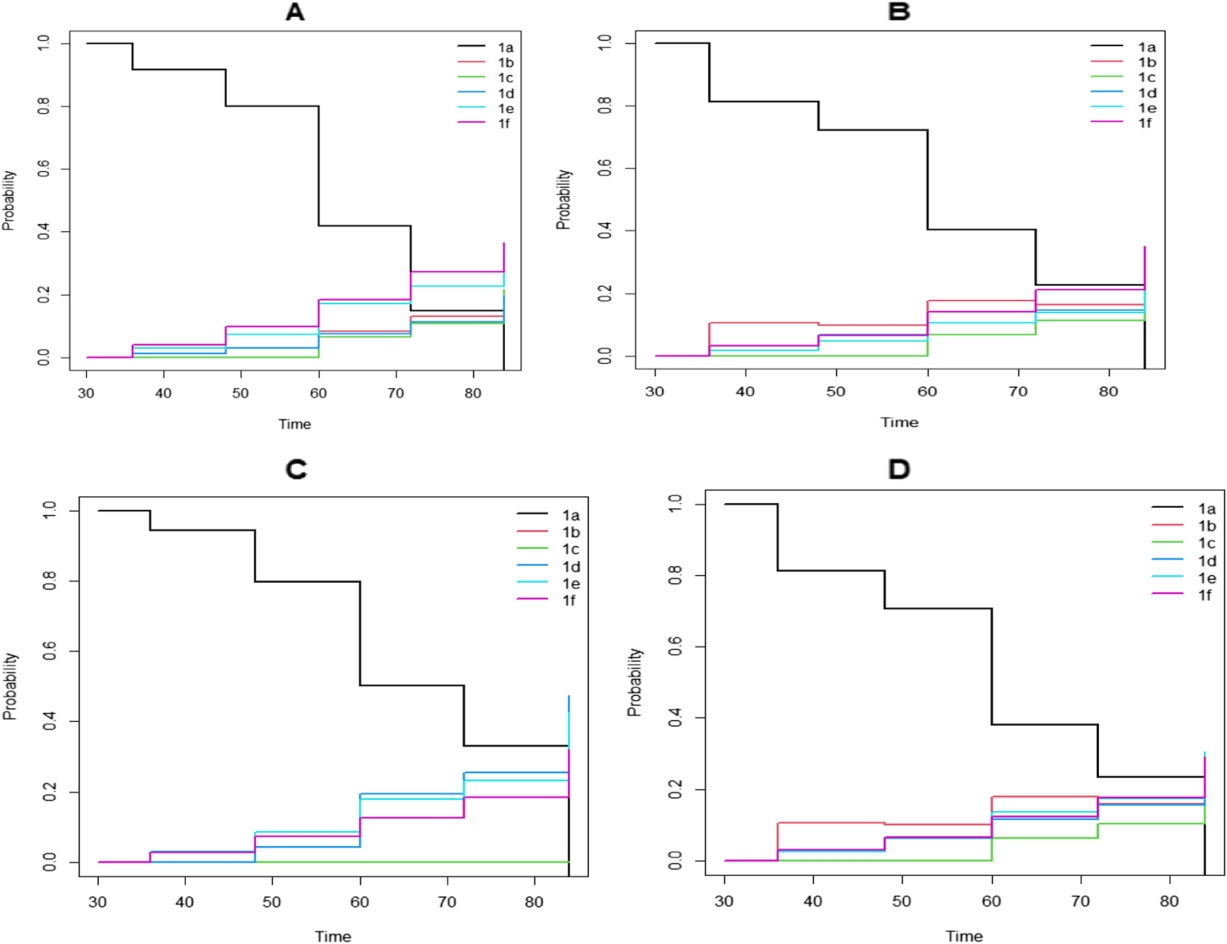
Prediction probabilities at s = 30 for a reference patient. **A** Probability of no treatment modification, **B.** Probability of NRTI substitution, **C.** Probability of NNRTI substitution and **D**. Probability of regimen changes. 1a: d4T-3TC-NVP, 1b: d4T + 3TC + EFV, 1c: AZT + 3TC + NVP, 1d: AZT + 3TC + EFV, 1e: TDF + 3TC + EFV, 1f: TDF + 3TC + NVP

In order to show predicted probabilities, we used the first six states of first line of treatment combinations, with a common set of covariates (age, weight, sex, tb status cd4 count) values. Prediction at some point after transition from one state to another state of patient with the given set of predictors (covariates) and given set of post events depends on the result of multi-state model. The predicted probability shown Fig. 5, as expected from different literature and also our above discussions, at initial time s = 0 and the state 1 & 2 (d4T) combination decrease the probability as the time passed 24 month and had the lowest staying probability means have highest probability of modifying their treatment combination as compared to(AZT or TDF), started increase probability after 24 months means more likely higher staying without modifying treatment than the other states. The NRTI substitution had greater on d4T initially and decreased the treatment modification but the other started increase the probability and lowest treatment modification. Early ART initiation, patient on AZT or TDF were treatment modification but patient on d4T lowest treatment modification and started worst as the time went up on follow up as compared to AZT or TDF. Similar result, in Fig. 6, as the time goes up, the prediction at s = 30 months, patient on d4T had good status on treatment combination all four Fig. 6(A, B, C, D) as compare to AZT or TDF in these figure but after the time went AZT or TDF had lowest treatment modification than patient on d4T combination. Comparing Fig. 5 & 6, early the ART initiation d4T were better but AZT or TDF started to increase probability means lowest treatment modification.

#### Factors associated with treatment switch

A cox-ph model, factors associated with a greater risk of switching was analysed using statistical R software language with package msm used to perform the multi-state analysis to obtain the effects of covariates on the transition intensities. The assumptions were tested and valid using three types of diagnostics for the Cox model (testing the proportional hazards assumption, examining influential observations (or outliers) and nonlinearity). Univariate multi-state models were built with statistical significance covariates. Covariates that showed statistical significance (p-value < 0.05) in the univariate analysis were considered in the final model. Hazard ratios (95% confidence intervals) of each transition are shown in Table 5 below.

**Table 5:** Hazard ratios and 95% confidence intervals for all covariates and all transitions

	**1a- > 1f**	**1c- > 1d**	**1e- > 1f**	**1f- > 1e**
	**exp(coef) CI 95%** ***p*-value**	**exp(coef) CI 95%** ***p*-value**	**exp(coef) CI 95%** ***p*-value**	**exp(coef) CI 95%** ***p*-value**
Sex				
Male	**Reference**	**Reference**	**Reference**	**Reference**
Female	**0.27(0.2–3.5) 0.44**	**2.4(0.85–3.83) 0.12**	**1.93(0.67–2.01) 0.98**	**0.74(0.3–0.1.02) 0.36**
Age	0**.99 (0.96–0.99) 0.98**	1.005**(0.98–1.03) 0.72**	0**.99 0.96–1.02) 0.98**	**0.99(0.96–1.07) 0.61**
Wt0	**1.1(1.01–1.12) 0.066**	**1.03(0.98–1.23) 0.39**	**1.03(1.001–1.05) 0.056**	**0.96(0.92–0.99) 0.0367**
Cd4 at baseline				
< = 200		**Reference**	**Reference**	**Reference**
201–350		**1.09 (0.36–1.25) 0.83**	**0.63(0.54–1.46) 0.25**	**1.24(0.34–2.31) 0.43**
351–500		**0.66(0.17–2.44) 0.61**	**1.59(0.94–3.36) 0.37**	**1.98(0.65–2.39) 0.17**
> = 501		**0.71(0.34–5.42) 0.6**	**0.71(0.76–2.84) 0.6**	**5.45(0.73–9.11) 0.25**
TB status				
Negative	**Reference**	**Reference**	**Reference**	**Reference**
Positive	**9(2.5–11.7) 0.0348**	**4.9(3.6–8.9) 0.0297**	**3.99(1.46–8.23) 0.0003**	**1.62 (0.35–4.78) 0.147**
**Anaemia**				
Normal		**Reference**	**Reference**	**Reference**
Anaemic		**2.29 (0.75–3.05) 0.091**	**1.44(0.79–2.20)0.27**	**1.18 (0.71–3.89)0.52**
OI_cat				
** No**		**Reference**	**Reference**	**Reference**
** Yes**		**1.06 (0.73–2.44) 0.87**	**2.13 (1.03–2.38) 0.0415**	**1.12 (0.67–3.46)0.65**

The risk of treatment modification was 9(2.13) 0.0348 times higher among TB positive patients at baseline compared to negative patients in this transition from 1a to 1f. Patients who have positive TB status were 4.9(0.73) 0.0297 times higher risk of modifying treatment from 1c to 1d compared to negative patients. Patients who have opportunistic infection at baseline have risk of treatments switching medication from 1e to 1f were 2.13 (0.37) 0.0415 times higher as compared to patients without opportunistic infection. Patients who have positive TB status were 3.99(0.38) 0.00029 treatments switching medication from 1e to 1f compared to TB negative patients. As the patient weight increases the transition of patient from medication combination 1f to 1e were 0.96(0.015) 0.0367 higher than those who have lower weight.

## Discussion

In the present study, we have considered a national dataset that include all regional states and the two administrative cities and thus, it can contributes evidence heading to national health policy. It could be considered as an extension from the previous few studies already made it in only one region and health centre. From 2019 to 2020 patients under ART, The mean age were 37.8 ± 10.37 (95%CI: 37.7, 37.9) years. Majority of the patients were around average age between 21 to 40 years old 20,723(63.47%) this result is higher as compare to a study in Nejo hospital in west Ethiopia [24] the reason might be this study covered the whole country but the comparison study was in one hospital only. Approximately 14.4% had modified their ART treatments, of which 13.26% were modified their treatments only once and 1.14% had modified their treatments more than once, which is similar to the study conducted in Switzerland [25], in Thailand[26], and smaller by far as compared to study in Ethiopia [9, 27], in Brazil [7] and Kenya [28]. Most treatment modification were done on the d4T combination and the median staying before modification were 24 month, AZT combination median staying before modification were 42 month and 36 month of TDF, this means combination treatment with d4T drug had high probability of modification which is almost smaller by half as compared to the study conducted in south Ethiopia [9, 27]. In this cohort, majority of the patients were on the TDF-based regime of TDF/3TC/EFV approximately (78.44%) and the remaining were the AZT/3TC/NVP (8.83%), TDF + 3TC + NVP (6.56%).

The important evidence provided us the reasons treatment modification were Toxicity/other side effect and commodity, drug out of stock, virology failure and immunological failure were 22.5%, 34.19%, 7.9%, 0.7%, and 2.43% respectively. These result is lower than the previous study [6, 9, 27] the difference might be, this study were national wide but the comparison study were conducted only one health facility and the other is the patient good adherence to their medication. The other interesting evidence brought in this study was estimation of the transition matrix, occupational and length of stay in each state. The transition states 1c- > 1e were (10.39%), 1a- > 1c were (5.28%) and 1c- > 1f were 5.69%. This study was higher as compared to the study conducted in south Ethiopian region [9, 29].

Also estimation transition probability and prediction were determined. The transition probability decrease means for those higher treatment modifications as the follow-up month increased for those treatment combination d4T but increase for those patients took treatment combination AZT or TDF. Similarly higher length of stay as the follow up time increase on treatment combination AZT or TDF as compare to d4T. Prediction pprobability at s = 0 months for a reference of patient, probability of no treatment modification and Probability of treatment modification, early the ART initiation d4T were better but AZT or TDF started to increase probability means lowest treatment modification and similarly the prediction probability at s = 30 the treatment combination d4T were worst treatment modification but AZT or TDF is lowest treatment modification. This result is in line with the result in the previous [9]. Finally, In this cohort, risk factors for patients modified their treatment in state 1a- > 1e were positive TB status, the risk of transition 1c- > 1d were positive TB status and those had opportunistic disease. Also as weight increase the risk of modifying or transition 1f- > 1e. This was similar to the study in south Ethiopia [29].

## Conclusion

This study shows the common reasons of treatment modification patient on ART some of them were toxicity/ comorbidity, immunological failure, viral load and drug not availability which accounts more than 50%. One of the combination d4T drugs a major apprehension modification of or regime change. The other important findings were the transition probability and occupational probability, as the follow-up time increase the transition probability increase and occupational probability decrease. Again the prediction probability at some point of follow-up time the patient had higher probability of modifying their treatment because of drug toxicity, comorbidity, drug not available and others. Removing the d4T drug combination with high toxicity profile, could reduce treatment switching and progress patient staying on the same state and stability of patient treatment.

### Supplementary Information

Supplementary Material 1.

## Data Availability

The data sets analyzed in this study available from the corresponding author on reasonable request. The R code used to analyze the data provided as a supplement of the article.

## Abbreviations

ART: Antiretroviral therapy
HAART: Highly active antiretroviral therapy
AHRI: Armauer Hansen Research Institute
AIDS: Acquired Immunodeficiency Syndrome
CD4: Cluster of Differentiation 4 or Classification Determinant
FHAPCO: Federal HIV/AIDS Prevention & Control Office
HIV: Human Immunodeficiency Virus
NAPES: National ART Program Effectiveness Study
IRB: Institutional Review Board
