# Supplementary material for: Multi-state Markov model for time to treatment changes for HIV/AIDS patients: a retrospective cohort national datasets, Ethiopia
Source: BMC Infect Dis. 2024 Jun 24;24:627. doi: 10.1186/s12879-024-09469-9 (PMC11194888; doi:10.1186/s12879-024-09469-9)
Supplement: Supplementary file 1 — Supplementary Material 1. [file 12879_2024_9469_MOESM1_ESM.docx]

Annex1.

1. Overall survival Null model


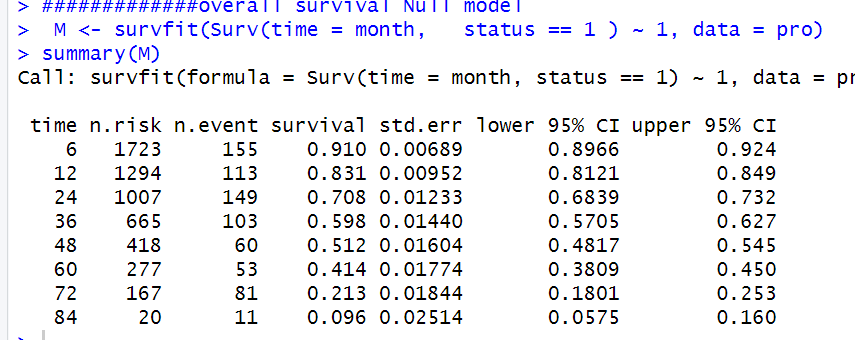


1. Overall survival model by sex


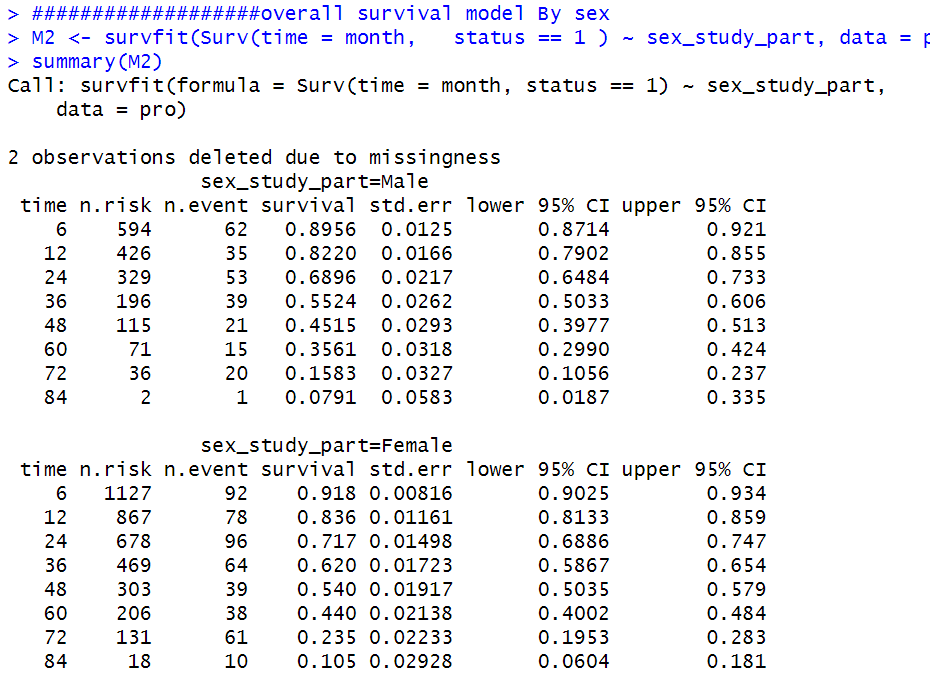


######################cox regression

m1<-coxph(Surv(start,stop,status) ~ age2 + wt0 + sex + anemia_0 + tb02+cd4_cat+ OI_cat, data = pro, subset = c(reg =="1a->1e"))

m1<-coxph(Surv(start,stop,status) ~ age2 + wt0 + sex + anemia_0 + tb02+cd4_cat+ OI_cat, data = pro, subset = c(reg =="1c->1d"))

m1<-coxph(Surv(start,stop,status) ~ age2 + wt0 + sex + anemia_0 + tb02+cd4_cat+ OI_cat, data = pro, subset = c(reg =="1e->1f"))

m1<-coxph(Surv(start,stop,status) ~ age2 + wt0 + sex + anemia_0 + tb02+cd4_cat+ OI_cat, data = pro, subset = c(reg =="1f->1e"))

####################transition probability

library(msm)

library(mstate)

library(dynpred)

library(mstate)

library(mstate)

library(dynpred)

library(survival)

library(survminer)

library(foreign)

library(ggplot2)

library(rms)

library(pec)

library(xfun)

library(Hmisc)

library(prodlim)

library(mstate)

pro<-read.dta("C:/Users/hp/Desktop/Mnuscript one and two 642023/newdata2.dta")

####################transition probability

head(pro)

table(pro$reg0)

M2 <- survfit(Surv(time= followuptime, status ) ~ 1, data = pro)

summary(M2)

plot(M2)

ggsurvplot(M2, data = pro, risk.table = TRUE, xlab="Time in month", lwd=3, linetype = c(1,2,3,4,5,6), pval = TRUE, ylab = c("probability"))

#####################figure 6

M2 <- survfit(Surv(time = month, status == 1 ) ~ initialstate, da = pro)

summary(M2)

plot(M2)

ggsurvplot(M2, data = pro, linetype = c(1,2,3,4,5,6,7,8,9), pval = TRUE, ylab = c("staying probability"))

M3 <- survfit(Surv(followuptime, status) ~ sex, data = pro)

summary(M3)

plot(M3)

ggsurvplot(M3, data = pro, risk.table = TRUE, xlab="Time in month", lwd=3, linetype = c(1,2,3,4,5,6), pval = TRUE, ylab = c("probability"))

ggsurvplot(M3, data = pro, linetype = c(1,2,3,4,5,6,7,8,9), pval = TRUE, ylab = c("staying probability"))

#########

pro<-read.dta("C:/Users/hp/Desktop/working data/work_22.dta")

head(pro)

M4 <- survfit(Surv(month, status) ~ substitute, data = pro)

M4 <- survfit(Surv(time = month, status == 1 ) ~ reg0, da = pro)

summary(M4)

plot(M4)

ggsurvplot(M4, data = pro, risk.table = TRUE, xlab="Time in month", lwd=2, linetype = c(1,2,3,4,5,6), pval = TRUE, ylab = c("probability"))

M5 <- survfit(Surv(time = month, status == 1 ) ~ reg, da = pro)

summary(M5)

plot(M5)

ggsurvplot(M5, data = pro, risk.table = TRUE, xlab="Time in month", lwd=1, linetype = c(1,2,3,4,5,6, 7,8,9), pval = TRUE, ylab = c("probability"))# Prediction

summary(m1)


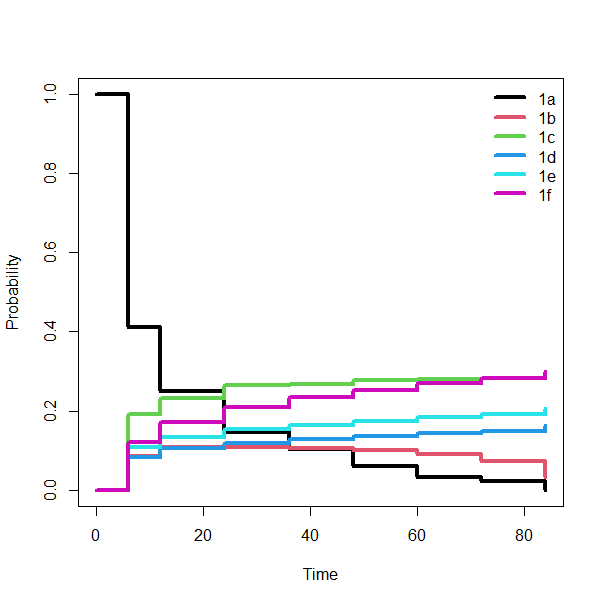

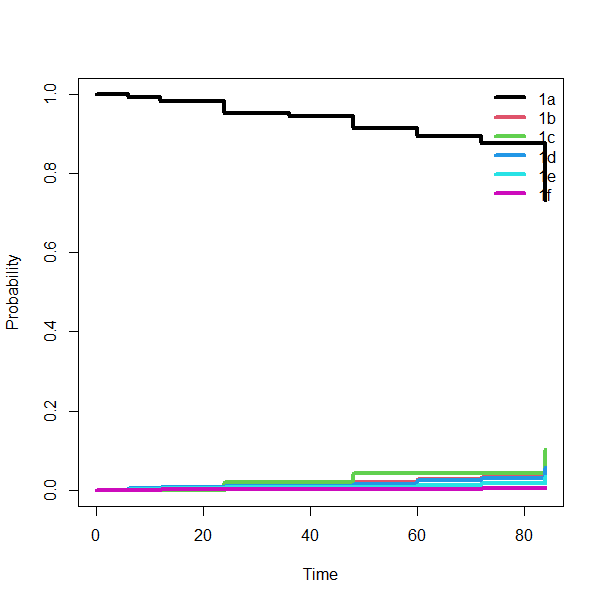


No treatment modification Treatment modification

Figure 1 follow up time 0


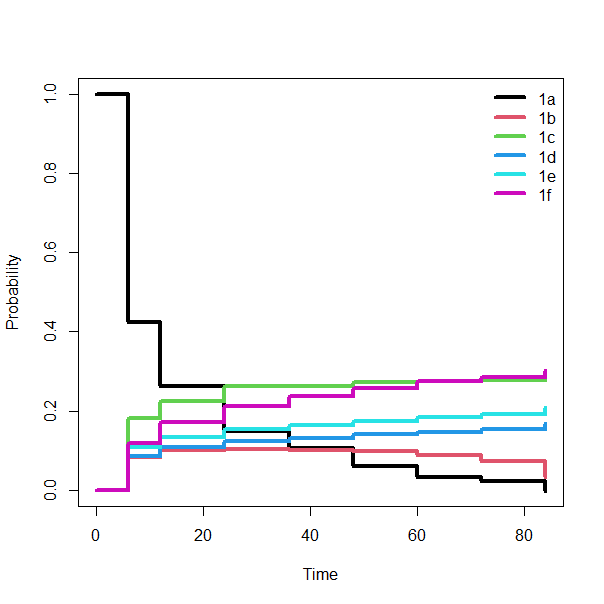

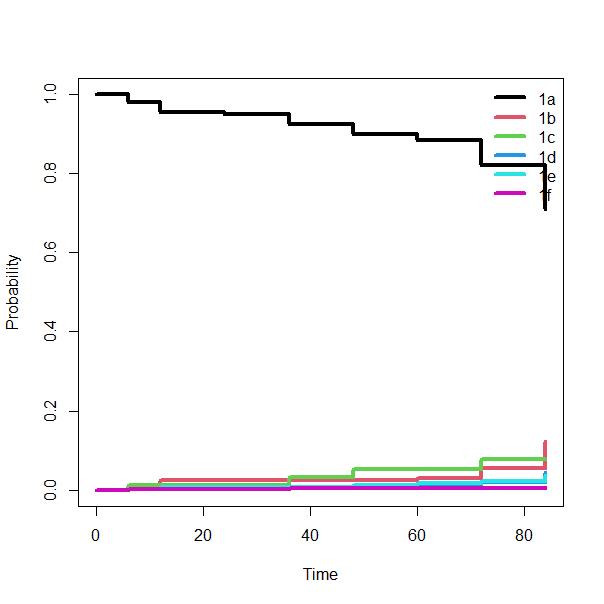
 Figure 1 follow up time 0


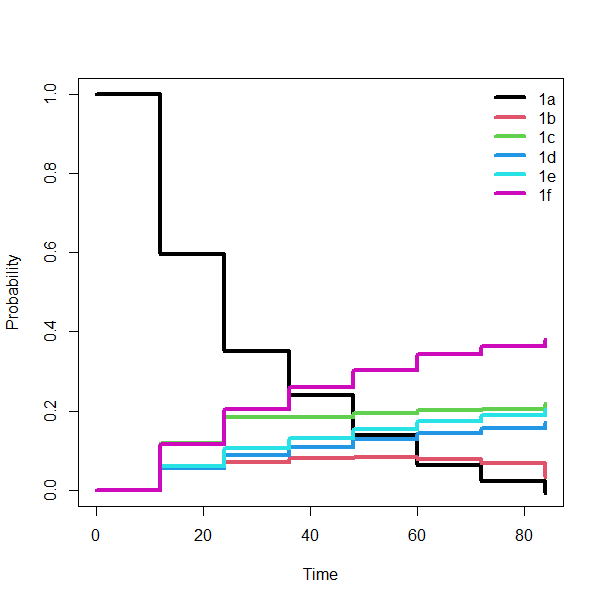

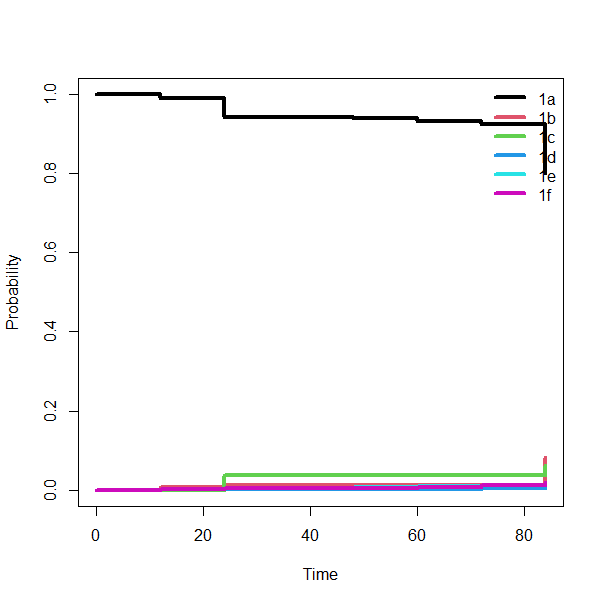


Figure 3 follow up time 12


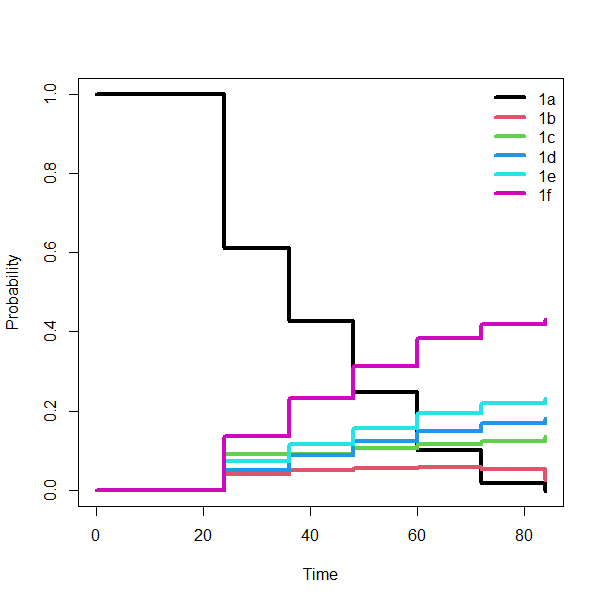

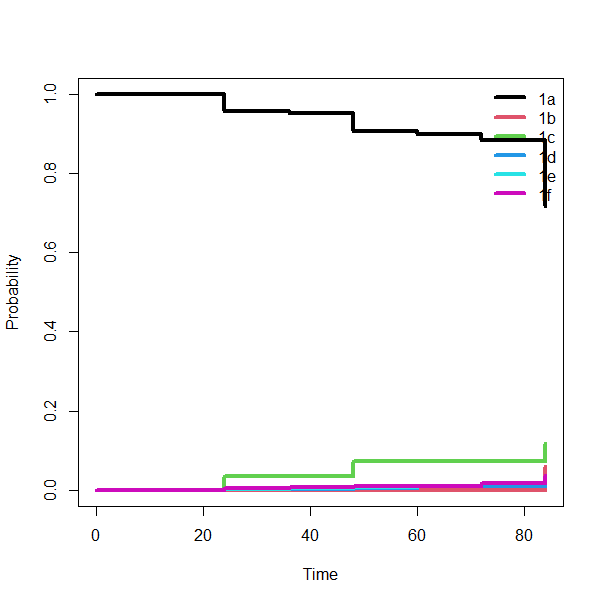


Figure 4 follow up time 24


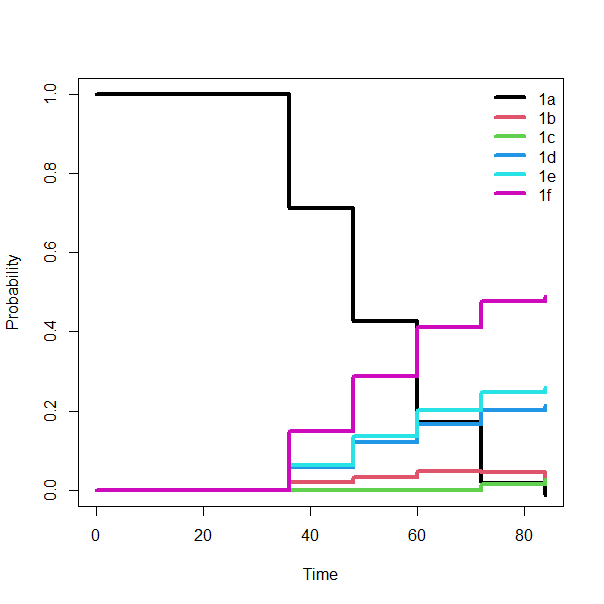

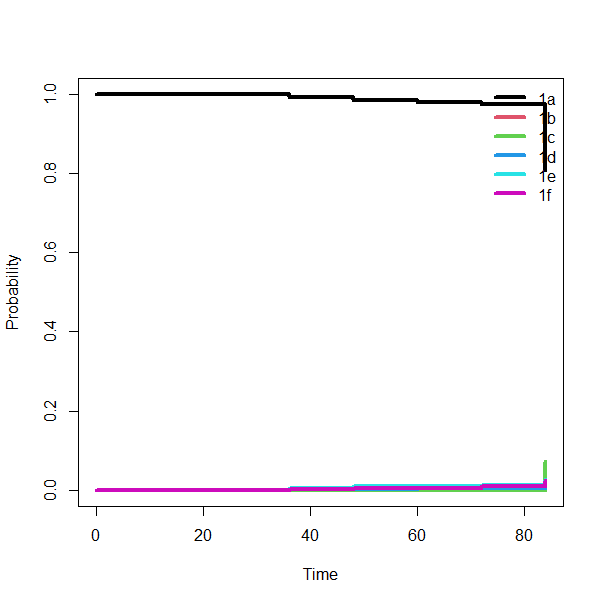


Figure 5 follow up time 36


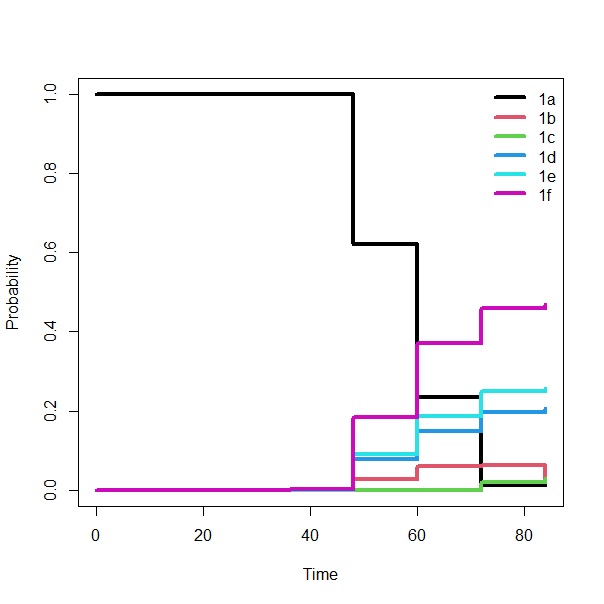

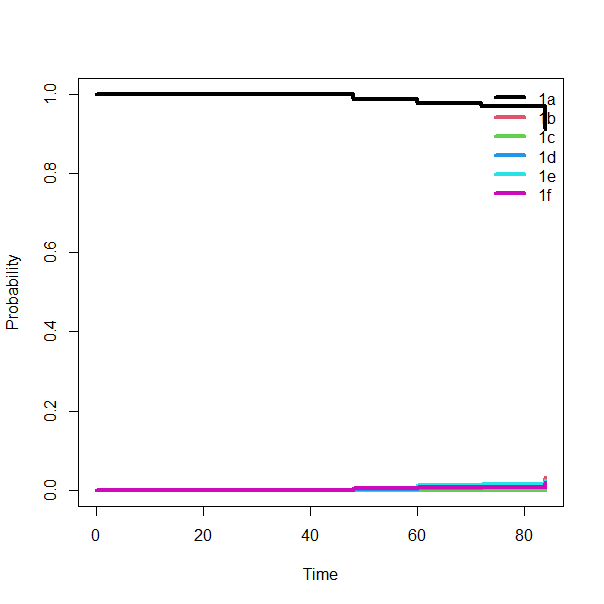


Figure 5 follow up time 48
